# ANOVA-HD: Analysis of variance when both input and output layers are high-dimensional

**DOI:** 10.1371/journal.pone.0243251

**Published:** 2020-12-14

**Authors:** Gustavo de los Campos, Torsten Pook, Agustin Gonzalez-Reymundez, Henner Simianer, George Mias, Ana I. Vazquez

**Affiliations:** 1 Epidemiology & Biostatistics, Michigan State University, East Lansing, MI, United States of America; 2 Statistics & Probability, Michigan State University, East Lansing, MI, United States of America; 3 Institute for Quantitative Health Science and Engineering, East Lansing, MI, United States of America; 4 Department of Animal Sciences, Center for Integrated Breeding Research, University of Goettingen, Goettingen, Germany; 5 Genetics and Genome Sciences Graduate Program, Michigan State University, East Lansing, MI, United States of America; 6 Biochemistry and Molecular Biology, Michigan State University, East Lansing, MI, United States of America; University of Sao Paulo/Luiz de Queiroz Agriculture College, BRAZIL

## Abstract

Modern genomic data sets often involve multiple data-layers (e.g., DNA-sequence, gene expression), each of which itself can be high-dimensional. The biological processes underlying these data-layers can lead to intricate multivariate association patterns. We propose and evaluate two methods to determine the proportion of variance of an output data set that can be explained by an input data set when both data panels are high dimensional. Our approach uses random-effects models to estimate the proportion of variance of vectors in the linear span of the output set that can be explained by regression on the input set. We consider a method based on an orthogonal basis (Eigen-ANOVA) and one that uses random vectors (Monte Carlo ANOVA, MC-ANOVA) in the linear span of the output set. Using simulations, we show that the MC-ANOVA method gave nearly unbiased estimates. Estimates produced by Eigen-ANOVA were also nearly unbiased, except when the shared variance was very high (e.g., >0.9). We demonstrate the potential insight that can be obtained from the use of MC-ANOVA and Eigen-ANOVA by applying these two methods to the study of multi-locus linkage disequilibrium in chicken (*Gallus gallus*) genomes and to the assessment of inter-dependencies between gene expression, methylation, and copy-number-variants in data from breast cancer tumors from humans (*Homo sapiens*). Our analyses reveal that in chicken breeding populations ~50,000 evenly-spaced SNPs are enough to fully capture the span of whole-genome-sequencing genomes. In the study of multi-omic breast cancer data, we found that the span of copy-number-variants can be fully explained using either methylation or gene expression data and that roughly 74% of the variance in gene expression can be predicted from methylation data.

## Introduction

Modern genomic data often combine information from multiple data-layers, each of which itself can be high-dimensional. Examples of this include data sets comprising of information from several omics, or those combining genomic information with high-throughput phenotyping (e.g., crop-imaging, milk infrared spectra data). The biological processes underlying each of the data-layers can induce complex dependencies between features within each layer (e.g., linkage disequilibrium among single nucleotide polymorphisms, SNPs) as well as between layers (e.g., the association between DNA and gene expression, GE). The main goal of this study is to develop and to evaluate methods to quantify multivariate-associations in settings in which both the input and output sets are high dimensional.

The methods proposed in this study can be used to answer ubiquitous questions such as: How much of the inter-individual differences in whole-genome sequence genotypes can be predicted using a low-density SNP array? What proportion of variance in GE can be explained by differences in DNA methylation (ME)? How much of the variance in image-derived phenotypes can be predicted from DNA genotypes?

Canonical Correlation Analysis (CCA, [1]), Multivariate-Analysis of Variance (MANOVA, [2]) and Reduced Rank-Regressions, (e.g., Partial Least Squares, PLS, [3]) are three methodologies often used to assess associations in multi-dimensional problems. However, these approaches have limitations that make some of them inadequate for estimating the proportion of variance explained when both the output and input layers are high-dimensional.

Canonical Correlation Analysis extends the concept of the correlation between two random variables to a multivariate context. However, correlation is symmetric by nature. Therefore, CCA cannot address questions regarding the proportion of variance explained when the proportion of variance of one set (e.g., ***X***) that is explained by another set (***W***) is not equal to the reciprocal (i.e., the proportion of variance of ***W*** that can be explained by ***X***). Many multi-layered data sets are not expected to have a symmetric variance-decomposition (we will illustrate this using simulated and experimental data).

Multivariate Analyses of Variance (MANOVA, [4]) is often used for ANOVA when both the response and the explanatory data sets are multi-dimensional. However, MANOVA is based on least-squares projections; therefore, the methodology is not well-suited for cases when data is high dimensional, including rank-deficient cases. Most of the problems that we focus on involve high-dimensional data where the number of features exceeds sample size; thus, making least-squares methods such as MANOVA inadequate.

Reduced-rank regressions [5] and penalized multivariate analysis methods [6] are often used to analyze high-dimensional data. However, the results that one can obtain using regularized methods rely on regularization decisions (e.g., the number of dimensions used in PLS or CCA, or the sparsity parameters in sparse CCA) which cannot be made using fitness (e.g., the likelihood function) or lack-of-fit measures (e.g., residual sum of squares) evaluated in the training data. Thus, these parameters are often tuned to maximize prediction accuracy in testing sets. However, solutions derived by maximizing cross-validation prediction accuracy are not necessarily optimal for inferences because prediction accuracy is highly dependent on the relationship between sample size (*n*) and model complexity (e.g., number of parameters, *p*). Thus, in cases where *p>>n*, optimal prediction accuracy may be achieved with a highly parsimonious model (e.g., a principal component regression based on a few axes) that can produce severely biased estimates of effects. Therefore, to overcome the limitations of existing methods, in this study, we developed and evaluated approaches for estimating the proportion of variance explained when both the input and output sets are high-dimensional.

## Results

We developed two methods that use random-effects models to estimate the proportion of variance of independent vectors in the linear span of an output layer that can be explained by regression on an input layer. We considered two approaches for generating a sequence of independent vectors in the linear span of the output layer: A Monte Carlo method (MC-ANOVA) which uses random vectors, and one based on eigenvectors (Eigen-ANOVA).

### Setting the stage

Consider a data set consisting of two numeric matrices, ***X***_*n*×*p*_ and ***W***_*n*×*q*_, holding data for *n* individuals (rows) and *p* (***X***) and *q* (***W***) features in columns, respectively. For instance, ***X*** may be a matrix with genotype codes at *p* SNPs and ***W*** may be a matrix providing GE levels assessed at *q* genes. The columns of ***X*** = {***x***_1_,***x***_2_,…,***x***_*p*_} and of ***W*** = {***w***_1_,***w***_2_,…,***w***_*q*_} can be viewed as axes spanning two linear spaces (*L*_*X*_ and *L*_*W*_, respectively). The linear spans (extensions to nonlinear settings will be addressed in the discussion) of ***X*** and ***W*** consist of all the vectors that can be obtained by forming linear combinations of the columns of each of these sets, that is $L_{X}=\{x_{s}:x_{s}=X\alpha_{s}=\sum_{j=1}^{p}x_{j}\alpha_{sj}\}$ and $L_{W}=\{w_{s}:w_{s}=W\delta_{s}=\sum_{j=1}^{q}w_{j}\delta_{sj}\}$, for all real-valued vectors ***α***_*s*_ = {*α*_*s*1_,…,*α*_*sp*_} and ***δ***_*s*_ = {*δ*_*s*1_,…,*δ*_*sq*_}. In the following, we will use ***W*** as the input set and ***X*** as the output set; however, the methods proposed are not symmetric and additional knowledge can be gained by switching the roles of ***X*** and ***W***.

For each vector ***x***_*s*_∈*L*_*X*_, one can estimate the proportion of variance that can be explained by linear regression on *L*_*W*_ using a model of the form

$$x_{s}=W\beta +\varepsilon .$$ (1)

For cases where *q* is large, the proportion of variance of ***x***_*s*_ that can be explained by regression on *L*_*X*_ $\left(R_{x_{s}}^{2}\right)$ can be estimated by regarding both ***β*** and ***ε*** as Gaussian independent random variables, $\beta \begin{array}{c} iid\\ ∼ \end{array}N(0,\sigma_{\beta }^{2})$ and, $\varepsilon \begin{array}{c} iid\\ ∼ \end{array}N(0,\sigma_{\varepsilon }^{2})$. Upon appropriate scaling of the columns of ***X*** (see Materials and Methods for details) $R_{x_{s}}^{2}=\frac{\sigma_{\beta }^{2}}{\sigma_{\beta }^{2}+\sigma_{\varepsilon }^{2}}$ can be interpreted as the proportion of variance of ***x***_*s*_ that could be explained by regression on the features included in ***W***. The variance parameters involved ($\sigma_{\beta }^{2}$ and $\sigma_{\varepsilon }^{2}$) can be estimated using Bayesian or Likelihood methods (e.g., restricted maximum likelihood, REML, [7]), as such methods were designed to handle common challenges of overfitting and collinearity in high dimensional data.

In the preceding paragraph we describe how one can estimate the proportion of variance of a vector in *L*_*X*_ (***x***_*s*_) that can be explained by regression on ***W***. Next, we generalize the idea to all vectors in *L*_*X*_. However, *L*_*X*_ contains an infinite number of vectors; therefore, some approximation is needed. Perhaps the most natural approach for estimating the proportion of variance of vectors in *L*_*X*_ that can be explained by regression on *L*_*W*_ is to regress each of the columns of ***X*** on ***W***. Such an analysis would produce a sequence of R^2^ estimates $\left\{R_{x_{1}}^{2},R_{x_{2}}^{2},\ldots ,R_{x_{p}}^{2}\right\}$, and the average R^2^, $R_{X∼W}^{2}=p^{-1}\sum_{s=1}^{p}R_{x_{s}}^{2}$, could be used to estimate the overall proportion of variance of ***X*** that could be explained by regression on ***W***. However, one limitation of this approach is that the columns of ***X*** are not necessarily independent. Many features may cluster (e.g., genes may be co-expressed, or SNPs may be in high linkage-disequilibrium) leading to groups of highly unbalanced sizes. When some features are highly-correlated, the simple average of individual R^2^-values may be driven by a few clusters of the columns of ***X***. Furthermore, when ***X*** is ultra-high dimensional (e.g., hundreds of thousands or million features) estimating $R_{x_{s}}^{2}$ (*s = 1*,*…*,*p*) one-feature-at-a-time will be computationally challenging. Therefore, to address these problems, we propose two methods that use independent vectors from the span of the output set; each of these methods are explained next.

### Monte Carlo analysis of variance (MC-ANOVA)

Since *L*_*X*_ is infinite, one cannot estimate $R_{x_{s}}^{2}$ for all vectors in *L*_*X*_. However, one can ‘explore’ the linear span of the output set by generating random vectors in *L*_*X*_ of the form ***x***_*s*_ = ***Xα***_*s*_, where ***α***_*s*_ is sampled from some distribution. This can be repeated for a large number of vectors in *L*_*X*_ to produce a sequence of estimates $\left\{R_{x_{s}}^{2}\right\}$, and the resulting sequence can be used to estimate the average proportion of variance explained as well as other features of the distribution of the sequence. The method is summarized in Box 1. Importantly, if ***α***_*s*_ and ***α***_*s*′_ are independent, so are ***x***_*s*_ and ***x***_*s*′_. Indeed, noting that ***X*** is not random and assuming that ***α***_*s*_ and ***α***_*s*′_ are sampled independently, we have that $p(X\alpha_{s},X\alpha_{s\prime })=p(X\alpha_{s}|X\alpha_{s\prime })p(X\alpha_{s\prime })=p(X\alpha_{s})p(X\alpha_{s\prime }|X)$; therefore, ***x***_*s*_ and ***x***_*s*′_ are independent.

**Box 1. Monte Carlo analysis of variance (MC-ANOVA)**

1. Draw a random vector ***α***_*s*_ from a proper multivariate distribution.
2. Form the linear combination ***x***_*s*_ = ***Xα***_*s*_.
3. Estimate the proportion of variance of ***x***_*s*_ $\left(R_{x_{s}}^{2}\right)$ using a random-effects model (expression [1]) with variance parameters estimated using either Bayesian or likelihood-type methods.
4. Repeat 1–3 *B* times (e.g., *B = 10*,*000*).
5. Use the sequence of estimated R-squared $\left\{R_{x_{1}}^{2},\ldots ,R_{x_{B}}^{2}\right\}$ to approximate the distribution of the $R_{x_{s}}^{2}$. An estimate of $R_{X∼W}^{2}$ can be obtained using the median or the average, $R_{X∼W}^{2}=B^{-1}\sum_{s=1}^{B}R_{x_{s}}^{2}$, R-sq. in the sequence.

In Box 1 we did not specify how the ***α***_*s*_ are generated; for this aspect of the algorihtm there are countless possibilities: weights can be sampled from distributions with continuous support (e.g., *p*-variate Gaussian) or from mixture models with a point of mass at zero. The weights may be independent or correlated, and the distributions may be symmetric or skewed. We will show later on (using data from chicken genomes) that the process used to generate the weights may affect some features of the distribution of the R^2^ values, albeit not necessarily the mean or the median R^2^. The possibility of using different processes for generating random vectors in *L*_*X*_ gives the MC-ANOVA a great deal of flexibility. For example, this method could be used to assess how the distribution of the proportion of variance explained may change for different trait architectures–we will further explore that flexibility in greater detail in one of the case studies presented below.

### Regression using orthogonal basis (Eigen-ANOVA)

An orthogonal basis for the row-space of ***X*** can be obtained from the singular-value decomposition of $X=U_{X}D_{X}V_{X}^{\prime }$, where ***U***_*X*_ and ***V***_*X*_ are the left- and right-singular vectors of ***X*** respectively, and ***D***_*X*_ is a diagonal matrix with the singular values of ***X*** in the diagonal. Both ***U***_*X*_ and ***V***_*X*_ are orthonormal, thus $U_{X}^{\prime }U_{X}=I$ and $V_{X}^{\prime }V_{X}=I$. Each vector in *L*_*X*_ can be represented as a linear combination of the left-singular vectors of ***X***. Therefore, our second method estimates the proportion of variance of each of the left-singular vectors of ***X*** that can be explained by regression on ***W***, and produces a global R^2^ estimate using a weighted sum of the R^2^ estimated for each singular vector (Box 2, note that $d_{i}^{2}$ and ***U***_*X*_ in Box 2 are also the non-zero eigenvalues and the eigenvectors of ***XX***′, respectively).

**Box 2. Eigen-ANOVA**

1. Generate an orthogonal basis for *L*_*X*_; for instance, compute the singular-value decomposition of $X=U_{X}D_{X}V_{X}^{\prime }$ where $U_{X}^{\prime }U_{X}=I$ and $V_{X}^{\prime }V_{X}=I$ form an orthonormal basis for the row- and column space of ***X*** respectively, and ***D***_*X*_ = *Diag*{*d*_*i*_} is a diagonal matrix with the singular values of ***X*** in its diagonal (*i = 1*,*…*,*r*, where *r* is the rank of ***X***).
2. Regress each of the left-singular vectors on *L*_*W*_ using a linear model such as that in expression [1] with ***u***_*i*_ = ***x***_*s*_, and estimate the proportion of variance of each vector that can be explained by regression on *L*_*W*_, $R_{u_{i}}^{2}$.
3. Estimate the global proportion of variance of vectors in *L*_*X*_ that can be explained by regression on *L*_*W*_ using $R^{2}=\frac{\sum_{i=1}^{r}d_{i}^{2}R_{u_{i}}^{2}}{\sum_{i=1}^{r}d_{i}^{2}}$.

### Statistical properties assessed via simulations

We evaluated the statistical performance of the two methods described above using simulated data panels with a known proportion of variance shared between input and output data set. We also compared the performance of the two proposed methods with that of the Partial Least Squares (PLS, [3])–a method commonly used to analyze high dimensional data. We considered two simulation settings. In both cases, the input set was obtained from a wheat (*Triticum*) genotype data set generated by the International Maize and Wheat Improvement Center (CIMMYT) which contains genotypes at 1,279 DNA-markers assessed in 599 wheat inbred lines (see Materials and Methods for further details on this data set).

We note here that while a genotype matrix contains strictly discrete values (0/1 or -1/1 for inbreed lines and 0/1/2 or -1/0/2 for outbred diploid individuals) the linear span of it includes vectors in R^n^. The vectors in the linear span of the genotypes can be thought as ‘breeding values’ formed as linear combinations of genotypes.

In our ***first simulation setting***, ***W***_599×1,279_ was the genotype matrix and ***X***_599×1,279_ was obtained by adding *iid* (independent and identically distributed) Gaussian noise to the genotype matrix. We tuned the variance of the noise to generate scenarios of the proportion of variance of ***X*** explained by ***W*** ranging from 0 (***X*** was pure noise) to 1 (***X = W***). For each simulated data set we then estimated the proportion of variance of ***X*** explained by regression of ***W*** using random-effects models, with variance parameters estimated using REML [7] (see Materials and Methods for details).

The Monte Carlo method estimated the proportion of variance of ***X*** explained by ***W*** without any noticeable bias (**Table 1**). However, the regression of the left-singular vectors of ***X*** on ***W*** in Eigen-ANOVA produced estimates that were downwardly biased in case the true proportion of variance of ***X*** explained by ***W*** was large (e.g., >0.5). Further inspection of the results for individual MC replicates suggested that the bias of the Eigen-ANOVA method was likely due to a relatively large number of ‘corner’ solutions (zero estimated proportion of variance) which were common for high-order eigenvectors (i.e., those with small eigenvalue)–we illustrate this in an analysis of multi-omic cancer data further below. The use of PLS led to a downwardly bias estimates in cases where the proportion of variance of ***X*** explained by ***W*** was low (0.1, 0.3, 0.5) and an upwardly biased estimates when proportion of variance explained was high (0.8, 0.9).

**Table 1:** Average (SD) estimate of the proportion of variance explained by simulation scenario (first column) and estimation method (simulation 1).

True proportion of variance explained	Estimates		
	Monte Carlo- ANOVA	Eigen-ANOVA	PLS
0.0	0.0082 (0.0028)	0.0081 (0.0006)	0.0017 (0.0001)
0.1	0.1002 (0.0083)	0.0983 (0.0019)	0.0478 (0.0034)
0.3	0.2991 (0.0108)	0.3020 (0.0028)	0.2412 (0.0075)
0.5	0.4992 (0.0102)	0.5054 (0.0028)	0.4865 (0.0076)
0.8	0.8006 (0.0055)	0.7857 (0.0017)	0.8451 (0.0036)
0.9	0.9012 (0.0033)	0.8685 (0.0011)	0.9403 (0.0016)
1.0	1.0000 (< .0001)	0.9377 (< .0001)	0.9988 (< .0001)

We then considered a ***second simulation setting*** to contemplate cases involving asymmetric proportion of variance explained. To achieve this, we formed ***X*** using a subset of the wheat marker genotypes (5%, 10%, 30%, 50%, 80%, 90%, 95%) and formed ***W*** by binding the columns of ***X*** with additional columns filled with *iid* Gaussian noise (***Z***), that is ***W***_599×1,279_ = [*X*_599×*p*_, ***Z***_599×(1,279−*p*)_] (*p<1*,*279*). The columns of ***X*** and ***W*** were centered and scaled to unit variance; therefore the share of the variance of ***W*** explained by ***X*** ($R_{W∼X}^{2}$) is known and equal to, $\frac{p}{1,279}$. Similarly, the proportion of variance of ***X*** explained by ***W*** ($R_{X∼W}^{2}$) is 1 because ***X*** is included in ***W***.

In our second simulation study, the MC-ANOVA method rendered nearly unbiased estimates of the proportion of variance of one set explained by the other (**Table 2**). However, the Eigen-ANOVA method and the PLS produced noticeable biases, with Eigen-ANOVA method again generating downwardly biased estimates in cases where the true proportion of variance explained was high, and the PLS generating downwardly (upwardly) biased estimates whenever the true proportion of variance was low (high).

**Table 2:** Average (SD) REML estimates of the proportion of variance explained by simulation scenario (first column) and estimation method (simulation 2).

Scenario	*X* regressed on *W*			*W* regressed on *X*		
$\frac{\#Columns\;of\;X}{\#Columns\;of\;W}$	MC-ANOVA	Eigen-ANOVA	PLS	MC-ANOVA	Eigen-ANOVA	PLS
0.05	0.9960 (0.0039)	0.9085 (0.0051)	0.8885 (0.0069)	0.0505 (0.0050)	0.0548 (0.0012)	0.0244 (0.0029)
0.10	0.9972 (0.0030)	0.8891 (0. 0041)	0.9193 (0.0036)	0.1000 (0. 0072)	0.1061 (0. 0018)	0.0652 (0.0038)
0.30	0.9964 (0.0025)	0.8835 (0.0024)	0.9781 (< .0001)	0.2999 (0.0106)	0.3060 (0.0028)	0.2656 (0.0068)
0.50	0.9943 (0.0028)	0.8989 (0.0019)	0.9954 (< .0001)	0.4996 (0.0102)	0.4977 (0.0030)	0.4902 (0.0072)
0.80	0.9965 (0.0013)	0.9223 (0.0010)	0.997 (< .0001)	0.8000 (0.0061)	0.7714 (0.0025)	0.8259 (0.0047)
0.90	0.9992 (0.0005)	0.9302 (0.0008)	0.9979 (< .0001)	0.9008 (0.0039)	0.8593 (0.0019)	0.9277 (0.0035)
0.95	0.9998 (0.0002)	0.9345 (0.0008)	0.9984 (< .0001)	0.9511 (0.0026)	0.9016 (0.0013)	0.9746 (0.0025)

### Applications to experimental data

We used the MC-ANOVA and Eigen-ANOVA to quantify the proportion of variance explained in two experimental data sets. The first one contains a set of ultra-high-density (UHD) SNPs from chicken (*Gallus gallus*) genomes derived from a combination of whole-genome sequencing (WGS) and imputation. We used this data set to assess the proportion of variance of UHD genotypes that can be captured and predicted using low-density SNP sets. The second data set involved three omic-layers (gene expression [GE], methylation [ME], and copy-number-variants [CNVs]) of human (*Homo sapiens)* female breast cancer patients. We used this data set to assess the proportion of variance at one omic that can be explained by another omic.

#### Quantifying multi-locus linkage disequilibrium between SNP panels

The continued reduction of genotyping and sequencing costs has led to a sustained increase in the number of loci that can be genotyped. In plant and animal breeding four typical genotyping options include customized low-density arrays with hundreds to a few thousand SNPs [8], commercial arrays of common SNPs with tens of thousands of SNPs [9], high-density SNP arrays with hundreds of thousands of SNPs [10, 11], and whole-genome sequence-derived SNP genotypes. The number of SNPs that can be derived from WGS varies between populations and sequencing depth but is usually of the order of tens of millions (UHD SNP genotypes). In recent years, several projects have produced large volumes of fully sequenced genomes for various agricultural species and model organisms. However, generating, storing, and fitting models with UHD-genotypes can be logistically, economically, and computationally challenging. Moreover, empirical evidence seems to suggest that using UHD SNP-genotypes does not lead to substantial gains in prediction accuracy relative to models trained using tens of thousands of SNPs [12–15]. This often leads to researchers wondering: *How many SNPs are needed to capture (almost all) the information contained in UHD SNP genotypes*? We used the MC- and Eigen-ANOVA methods to address precisely this question.

Data consisted of 1.79 million SNP-genotypes for 892 (female and male) chickens from six generations of a purebred commercial brown layer line of Lohmann Tierzucht GmbH. These genotypes originated from a combination of whole-genome sequencing of 25 layers and imputation to UHD of the genomes of 867 that were genotyped a high density (~600,000 SNPs) Affymetrix Axiom Chicken Genotyping Array [16]. Further details about this data set can be found in the Materials and Methods section.

In a first analysis, the output space was the linear space (*L*_*X*_) spanned by the UHD SNP genotypes. The input set, (*L*_*W*_), consisted of low-density genotypes obtained by selecting *p* (*p* = 500, 1K, 2K, 3K, 5K, 10K, and 50K) evenly-spaced (in variant counts) SNPs. We estimated the proportion of variance captured by low-density panels using the MC- and Eigen-ANOVA methods. For the MC method we sampled weights from *iid* standard normal distribution, $\alpha_{js}\begin{array}{c} iid\\ ∼ \end{array}N(0,1)$, and then formed a random vector in *L*_*X*_ using ***x***_*s*_ = ***Xα***_*s*_, where ***X*** is the matrix of UHD SNP-genotypes. These random vectors were then regressed on the lower-density SNP sets, and the proportion of variance explained was estimated using REML. This was repeated 1,000 times to estimate the distribution of the proportion of variance of vectors in *L*_*X*_ explained by each of the low-density SNP-sets. For the Eigen-ANOVA method, we regressed each of the left-singular vectors of the UHD SNP genotypes on the low-density panels.

**Fig 1:**
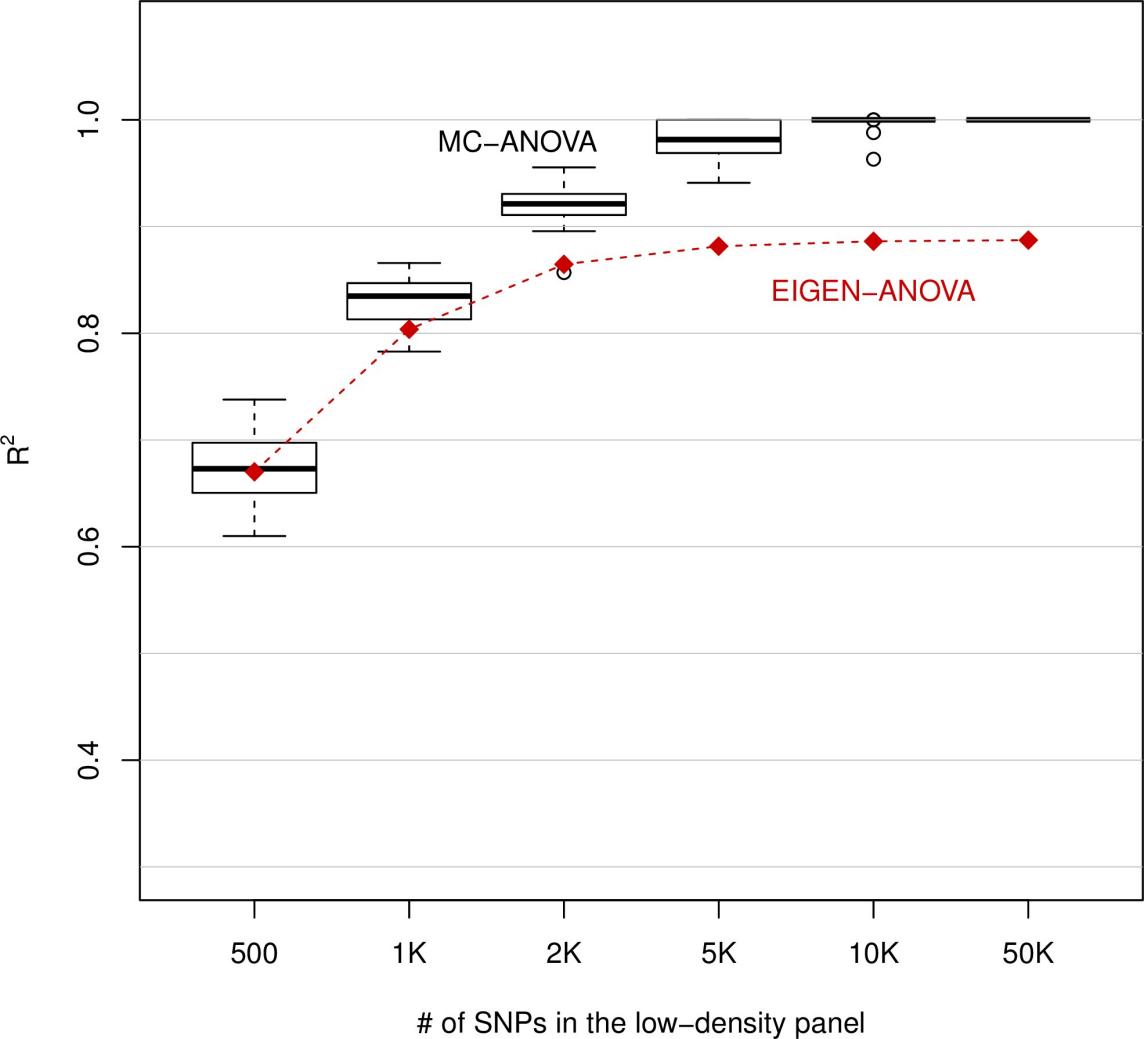
Proportion of the variance of whole-genome-sequence-derived SNPs (1.79 million) explained by SNP-panels consisting of 500, to 50K (K = 1000) evenly-spaced SNPs.

According to the MC-ANOVA method, the panel containing 500 evenly-spaced SNPs captured about two-thirds of the variance spanned by the UHD SNP genotypes (**Fig 1**). The proportion of variance of the UHD SNPs explained by low-density panels increased with the number of SNPs in the low-density panels reaching 100% with p> = 10K SNPs. The variance in the proportion of variance captured by low-density panels also decreased with the number of SNPs in the array (**Fig 1**). Small sample size and small effective population size are further factors that may make 10K SNPs to be sufficient to achieve a very high R-sq.

The Eigen-ANOVA yielded a very similar estimate of the proportion of variance explained as the MC-ANOVA for *p* = 500. However, for SNP-panels with more than 500 SNPs, the estimated proportion of variance obtained with the Eigen-ANOVA was systematically lower than the one obtained with MC-ANOVA. This agrees with what we found in the simulations where for high R^2^ the Eigen-ANOVA method gave downwardly biased estimates. (Note that while the MC-ANOVA yields both a point estimate and measures of dispersion (across random vectors) of R^2^, the Eigen-ANOVA only yields the point-estimates which are shown in **Fig 1**.)

In the previous application of the MC method, we drew random effect vectors that had weights (drawn from a normal distribution) on all the SNPs of the UHD set. When ***X*** contains whole-genome sequence genomes, one can think of the random vectors in *L*_*X*_ (***x***_*s*_ = ***Xα***_*s*_) as additive-genetic traits and of the MC method as exploring many possible of such traits. However, for any trait, the vast majority of variants in the genome are expected to have no effect. The number of variants affecting any trait could vary from very few (simple traits) to hundreds or thousands (complex traits). Therefore, to explore the effect of the trait architecture on the distribution of the proportion of genetic variance of those traits that could be captured by low-density SNP panels, we repeated the previous analyses using random vectors that had *5*,*10*,*50*,*500* non-zero weights–the set of SNPs with non-zero weight were randomly sampled from the UHD-genotypes, and the weights of those SNPs were *iid* normal (see Materials and Methods for details).

**Fig 2:**
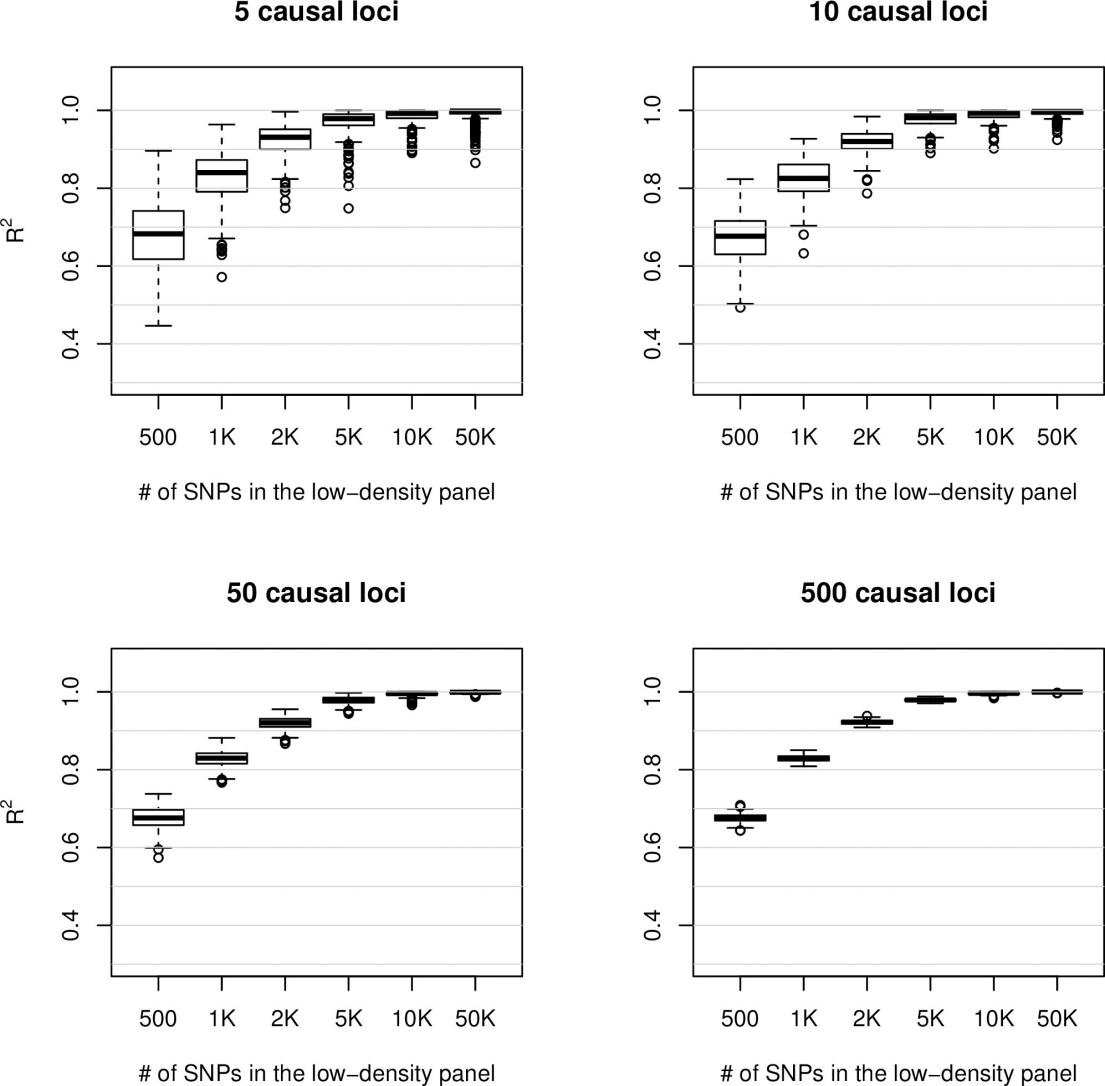
Proportion of variance of random vectors derived from ultra-high-density SNP-panel explained by regression on low-density SNP-panels, by number of loci used to form “genetic traits”.

The estimated proportion of variance explained by regression on lower-density SNP panels was, on average, the same across “trait-architectures” (**Fig 2**). However, the dispersion of the estimated means was, as expected, much larger for simple traits (e.g., 5 ‘causal variants’). For "complex traits" with 500 "causal variants," the proportion of variance explained by regression on 10K or more SNPs was greater than 95% for all MC replicates. However, for simpler traits we had some random vectors with a proportion of variance explained smaller than 0.8. This suggests that, while for highly complex traits low-density SNP arrays of 10K-50K SNPs may be enough to span the variance of the whole genome, for some simple traits, such arrays may not contain enough SNPs in high LD with the causal variants.

#### Using MC-ANOVA and Eigen-ANOVA to study shared variance in multi-omic data sets

Cancerous processes involve the deregulation of signaling pathways controlling cell fate and progression, arising from the accumulation of genomic and epigenomics alterations across multiple genes [17, 18]. Genetic and epigenetic modifications can lead to changes in GE, which in turn can lead to changes in downstream (e.g., protein expression) and upstream (e.g., DNA, ME) processes, thus resulting in complex multivariate association patterns between multiple omic-layers.

We used GE, ME and CNV data from breast cancer tumors (*n = 593*) from The Cancer Genome Atlas (TCGA) to study multivariate associations between those three omics. Details of the technologies used to generate these data, as well as the data quality controls (QC) and editions are described in the Materials and Methods. After QC and editions data consisted of the (log-transformed) expression of 20,319 genes, counts at 11,552 CVN-sites, and ME intensity at 28,241 ME CpG islands. We used the MC- and the Eigen-ANOVA methods to estimate the proportion of variance of one omic that can be explained by regression on another omic; we did this for all pairwise omics combinations (GE~ME, GE~CNV, ME~GE, ME~CVN, CNV~GE, and CVN~ME).

Our results with the MC-ANOVA method indicate that the CNV data were completely explained by both GE and ME (**Table 3**). About 70% of the variance spanned by ME was explained by GE and vice versa. Finally, CNV explained a relatively small fraction of the variance spanned by either GE or ME. These results suggest that most CNVs have effects in both ME and GE and therefore, variation in CNV can be predicted by ME and GE. However, although there is an association between CNV and both ME and GE, many other factors (e.g., environmental effects) seem to intervene, thus making the proportion of GE or ME explained by CNV relatively small (~20%). Overall the MC- and Eigen-ANOVA methods yielded similar results. However, in cases involving high R^2^ (CNV~ME, CNV~GE, GE~ME and ME~GE) the Eigen-ANOVA method gave R^2^ estimates that were lower than those of the MC method. This pattern is consistent with what we observed in the simulation and in the analyses of chicken genomes.

**Table 3:** Proportion of variance of one omic explained (posterior standard deviation) by regression of the omic in each row on the omic in each column obtained with MC-ANOVA (Eigen-ANOVA).

Dependent	Explanatory		
	CNV	Methylation	Gene Expression
CNV	---	1.00 (0.929)	1.00 (0.904)
Methylation	0.164 (0.228)	---	0.715 (0.685)
Gene Expression	0.204 (0.238)	0.738 (0.660)	---

**Fig 3:**
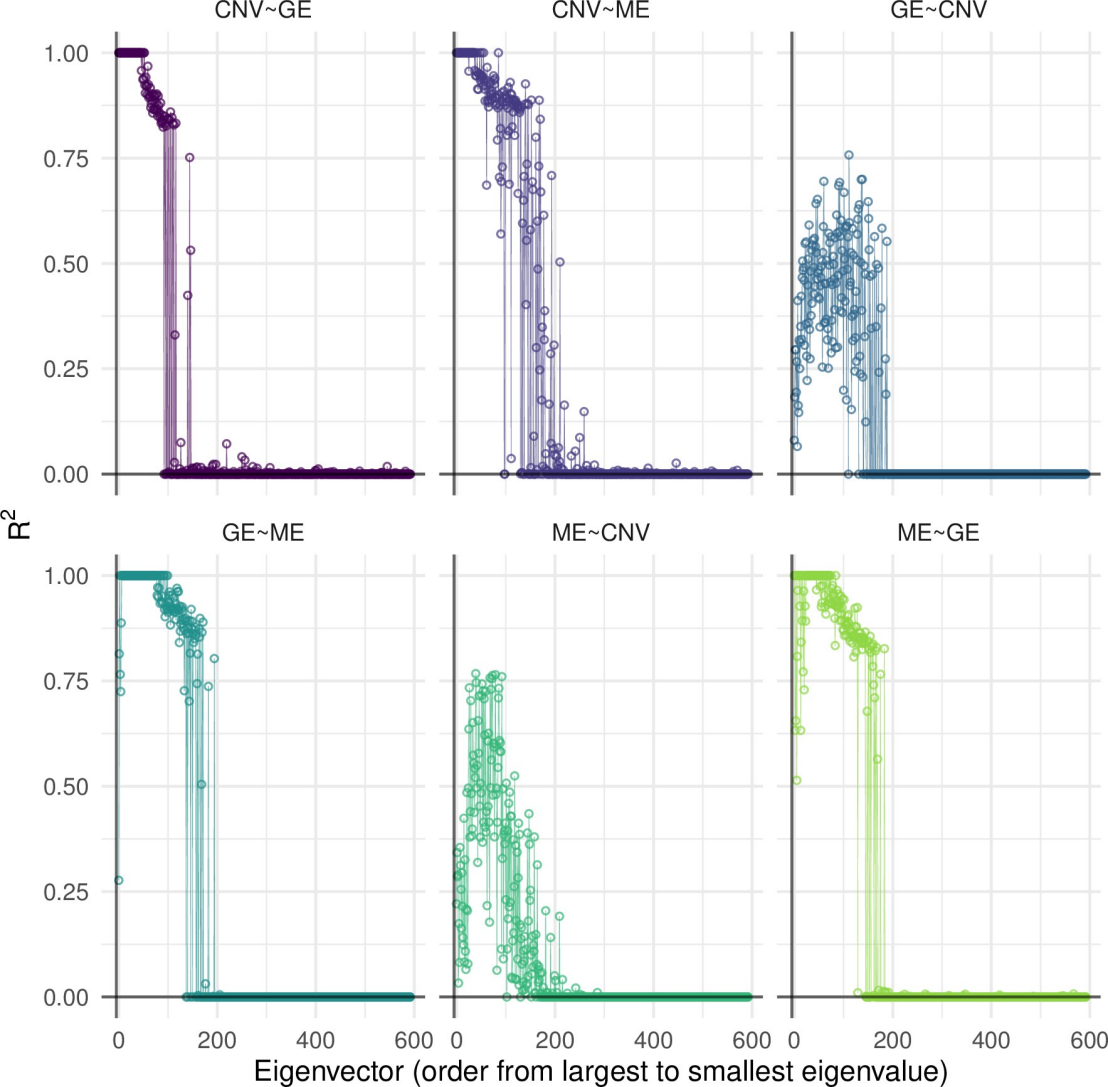
Proportion of variance of omic-derived eigenvectors of an omic-set explained by regression on a different omic-set. Points give the proportion of variance for individual eigenvectors. GE = Gene Expression, ME = Methylation, CNV = Copy-number variants (global R^2^ estimates, derived from random vectors and from the Eigen-ANOVA method are shown in **Table 3**).

Eigen-vector-specific R^2^ values obtained with the Eigen-ANOVA method (**Fig 3**) showed that the R^2^ values were, in most cases (except GE~CNV and ME~CNV) very high (and in many cases very close to one) for the top-eigenvectors (i.e., those with high eigenvalue), and very small for eigenvectors associated with low eigenvalues. The transition in the R^2^ profile of individual eigenvectors showed a relatively sharp phase transition from R^2^ values near one to near-zero values. Overall, our results suggest a relatively good agreement in the patterns captured by the top-eigenvectors across omics.

## Discussion

Modern genomic data sets often combine information from multiple non-independent data-layers. Quantifying multivariate associations between data layers can shed light on many important aspects of the data. In this study, we developed two procedures to estimate the proportion of variance explained in settings where both the input and output sets are high-dimensional. The proposed approach uses random effects Gaussian models to estimate the proportion of variance of (independent) vectors in the linear span of an output set (***X***) that can be explained by regression on an input set (***W***). The resulting R^2^ estimate is a weighted average of the R^2^ values obtained from independent vectors. To generate independent vectors, we considered two approaches: The first one (MC-ANOVA) is a Monte Carlo method that uses randomly generated vectors in the linear span of the output set. The second one (Eigen-ANOVA) uses an orthogonal basis for the linear span of ***X***.

The proposed methods share four important features. First, both methods can be used to perform analysis of variance when both explanatory and dependent data are high-dimensional. Second, estimates are entirely based on the likelihood function and there is no need to make regularization decisions (number of dimensions, penalty parameters). Third, for any pair of information sets, the analysis of variance is not necessarily symmetric; therefore, the approach accommodates cases where the proportion of variance of ***W*** explained by ***X*** is not equal to the reciprocal. Finally, in addition to producing an R^2^ estimate, the proposed methods can shed light on important aspects of the underlying association patterns (e.g., decomposition of the global R^2^ on eigen-vector specific R^2^’s, distribution of R^2^ over possible vectors in the linear span of the output set).

Our simulations suggest that MC-ANOVA renders nearly unbiased estimates of the proportion of the variance of one set that can be explained by another. However, the Eigen-ANOVA exhibited systematic biases in scenarios in which the true proportion of variance was high. We also evaluated the PLS regression method, and our simulations suggest that PLS lead to upwardly (downwardly) biased estimates whenever the true proportion of variance is high (low). Therefore, for estimation of the proportion of variance explained we recommend using MC-ANOVA. The Eigen-ANOVA method seems to be a valid alternative, provided that the proportion of variance of one set explained by the other is not too high.

### Computational considerations

The Eigen-ANOVA requires computing all the eigenvectors of the response matrix (say ***X***) and then estimating proportion of variance of each of the eigenvectors explained by the explanatory matrix (e.g., ***W***). The computational complexity of standard algorithms for singular-value decomposition is *O*(*n*^3^) (assuming *n~p*). On the other hand, the MC-ANOVA requires forming *B* random vectors fo the form ***x***_*s*_ = ***Xα***_*s*_; ignoring the cost of sampling the weights, the computational complexity of forming each of this vectors is *O*(*n*^2^), again assuming *n~p*; thus, in general the MC-ANOVA will be computationally less involved as long as the number of vectors required for accurate estimation is smaller than *n*. In our experience, a few hundred random vectors (say 300) are enough to estimate the average, median, and SD of the R^2^. Therefore, whenever the rank of the response matrix is high, the MC-ANOVA has clear computational advantages. These advantages would be particularly clear for very large rank matrices. Finally, we note that the estimation process of both Eigen-ANOVA and MC-ANOVA is 'embarrassingly' parallel since the R^2^ of each of the vectors (either eigenvectors or random vectors) can be computed independently of each other.

Consistent with our simulation results, the analyses of experimental data showed that in problems involving a high R^2^ the Eigen-ANOVA method yielded lower estimates of the proportion of variance explained than those obtained with the MC-ANOVA (e.g., see **Fig 1** and **Table 3**). Inspection of the results of the Eigen-ANOVA for individual eigenvectors suggests that the downward bias of the method may originate from ‘corner’ solutions (zero-estimates of R^2^) for eigenvectors associated with small eigenvalues. Therefore, if the only goal is to estimate the proportion of variance of one set explained by another set, we recommend using the MC-ANOVA method.

The Eigen-ANOVA method yields R^2^-values for each of the eigenvectors of the output set. This information can help elucidate whether global patterns (e.g., those associated with the top-eigenvectors) in one information set can be predicted from information contained in another information set. For instance, our analysis of the multi-omic breast cancer revealed that the patterns described in the top-eigenvectors derived from GE and ME are very similar; therefore, one should not expect big differences in tumor classifications that are based on the top-eigenvectors derived from either set. Interestingly, we found that in the analyses of omic data the R^2^ of individual eigenvectors showed a very sharp phase transition, suggesting that eigenvectors associated with intermediate and small eigenvalues may describe omic-specific patterns, or perhaps measurement error associated to each of the techniques.

The MC-ANOVA method can be used to characterize the distribution of the R^2^ estimates across vectors in the linear span of the output set. We used this feature to study the effect of the trait-architecture on the distribution of the R^2^ estimates. Our results indicate that while the average R^2^ does not seem to be affected by the sparsity of the coefficients used to form random vectors (i.e., the *α*_*s*_′), the dispersion and the shape of the distribution highly depend on the process used to generate the weights (Fig 2). Highly sparse weights lead to a distribution of the R^2^ values that, compared with vectors that were less sparse, had higher dispersion and in some cases (e.g., when the proportion of variance explained was close to 1) was skewed (Fig 2).

An important feature of the methods proposed in this study is that the R^2^ measure is not symmetric, in contrast to CCA. Our simulation study shows that if the underlying patterns are non-symmetric (e.g., when one of the linear spaces is a subspace of the other) the proposed estimation methods (in particular the MC-ANOVA) can detect the lack of symmetry very well (see **Table 2**). Interestingly, our analysis of multi-omic data from breast cancer patients exhibited cases where R^2^ was rather symmetric (e.g., the regression ME~GE and the regression ME~GE) and others that were highly asymmetric (e.g., CNV~GE and GE~CNV). The asymmetric cases suggest that almost all the variability in CNV can be predicted from GE (and ME as well); however, only a fraction of the GE variance can be explained by differences in CNV patterns. This result is consistent with the hypothesis that most CNV have an impact on GE, but GE is also affected by factors other than CNV (e.g., methylation, environmental effects).

In this study, we focused on the application of the Eigen- and MC-ANOVA for problems involving two input sets (e.g., a low- and a high-dimensional SNP array, or two different omics) evaluated on the same set of individuals. However, with slight modifications, the MC-ANOVA method will be useful for evaluating the proportion of variance of vectors in the span of a training set (e.g., all the available genotypes/phenotypes) that could be captured/predicted by regression on a subset of it, e.g., founders, or “proven” individuals, e.g., [19, 20].

The methods discussed in this study are entirely based on linear models. However, both MC-ANOVA and Eigen-ANOVA can easily be extended to consider non-linear relationships by embedding each set using a non-linear mapping. For instance, in the case of SNPs, one could generate a linear space that accounts for additive and non-additive effects by considering contrasts for additive, dominance, and epistatic interactions [21]. More generally, one can consider embedding either ***X*** or ***W*** by transforming one or both sets using a non-linear mapping *f*(.) (e.g., Gaussian kernels). Then, the methods presented here could be applied using $\tilde{X}=f(X)$ and $\tilde{W}=f(W)$ as information sets within the context of Reproducing Kernel Hilbert Spaces regressions (e.g., [22, 23]).

In our applications, we considered one dependent and one explanatory set; however, the methodology presented in this study could be easily adapted to accommodate cases with one output set (e.g., Y) and multiple explanatory sets (e.g., X, and W). This can be done by expanding the model used to estimate R^2^ [1] by including two random effects, each with its own variance parameter. Such methods could be used to answer potential questions such as what proportion of variance of gene expression may be explained by joint regression on methylation and copy-number-variants.

In conclusion, we developed two methods for estimating the proportion of variance explained in problems in which both the input and output sets are high-dimensional. The MC-ANOVA method provided nearly unbiased estimates across a range of simulation scenarios. In addition to providing estimates of the proportion of variance explained, the two methods can yield useful insight into the association patterns underlying multi-layered high-dimensional data.

## Materials and methods

### Data sets

The **wheat data set** used in **Simulations 1 and 2** was generated by the International Maize and Wheat Improvement Center (CIMMYT). This data set provides genotypes at 1,279 molecular markers (Diversity Array Technology DNA markers) assessed in 599 wheat inbred lines. Further details about this data set can be found in [24]. The data set is available with the BGLR R-package [25].

The **chicken data set** used in **Case Study 1** consisted of UHD SNP genotypes of 892 female and male chickens from six generations of a purebred commercial brown layer line of Lohmann Tierzucht GmbH. The genomes of 25 layers were sequenced at 8x read-depth. A total of 4.92M (M = million) SNPs were derived from these 25 genome sequences. The remaining layers (n = 867) were genotyped using the Affymetrix Axiom Chicken Genotyping Array [16] which contains ~600K (580,961) SNPs. Ni et al. [28] imputed the SNP-genotypes of those 867 layers to the whole-genome sequence (4.92 SNPs) using BEAGLE 3.3.2 [26] for phasing and MiniMac3 [27] for imputation. For details on the imputing procedure we refer to Ni et al. [28]. This produced a combined genotype file consisting of 4.92M SNPs from 892 = 25+867 genomes. We further filtered the combined genotype file by removing SNPs with minor-allele-frequency smaller than 0.005 (0.5%) and pruning adjacent SNPs that were in (almost) perfect LD (i.e., R^2^ ≥0.99). A total of 1.79M SNPs passed these last two filters.

The **breast cancer data set** used in **Case Study 2** was from the Cancer Genome Atlas (TCGA https://www.cancer.gov/tcga) and consisted of gene expression (GE), methylation (ME), and copy-number-variants (CNV) data from (n = 593) breast cancer tumors from female breast cancer patients.

Gene expression data (RNA-Sequencing counts) were generated using the Illumina HiSeq RNA V2 platform and DNA methylation profiles were determined using the Illumina HM450 platform. RNA-sequencing data were transformed using the natural logarithm and individual CpG site β-values were summarized at the CpG island level, using the maximum connectivity approach implemented in the WGCNA R package [29]. The CpG island summaries were transformed into M-values (M = β/(1-β) [30]). CNV profiles corresponded to gene-level copy number intensity derived from Affymetrix SNP Array 6.0 platform, using hg19 as reference.

From each of the three omics we removed features with a coefficient of variation smaller than 1% and those with a proportion of missing values greater than 20%. The missing values that remained were imputed using the clustering algorithm described in [31]. After imputation, each feature was adjusted for batch effects using ComBat [32]. After applying the steps described above, the data set used in the analyses consisted of the (log-transformed) expression of 20,319 genes, 11,552 CVN-sites, and ME intensity at 28,241 ME CpG islands.

### Restricted maximum likelihood estimation of variance components

For the **MC-ANOVA and EIGEN-ANOVA** methods, the proportion of variance of one set (e.g., ***X***) explained by the other set (***W***) was estimated using a random-effects model of the form

z=1μ+Wβ+ε,

where ***z*** was either a random linear combination of the columns of ***X*** (MC-ANOVA, see Box 1) or one of the eigenvectors of ***XX***′ (Eigen-ANOVA, see Box 2), *μ* is an intercept, ***β*** is a vector of Gaussian random effects, $\beta \begin{array}{c} iid\\ ∼ \end{array}N(0,\sigma_{\beta }^{2})$, and ***ε*** is a vector containing error terms, which were also assumed to be Gaussian, $\varepsilon \begin{array}{c} iid\\ ∼ \end{array}N(0,\sigma_{\varepsilon }^{2})$. For computational convenience and without loss of generality, we reparametrized the above model in terms of a random-effects model, of the form

z=1μ+u+ε,

where $u=W\beta ∼MVN(0,WW^{\prime }\sigma_{\beta }^{2})$, where *MVN*() stands for Multivariate Normal Distribution. We centered and scaled the columns of ***W*** to a standard deviation equal to $1/\sqrt{ncol(W)}$; this leads to a covariance structure, ***WW***′ with an average diagonal equal to one; therefore, with this scaling, $\sigma_{\beta }^{2}$ can be interpreted as the amount of variance of ***z*** captured by regression on ***W*** and the ratio $R_{z}^{2}=\frac{\sigma_{\beta }^{2}}{\sigma_{\beta }^{2}+\sigma_{\varepsilon }^{2}}$ can be interpreted as the proportion of variance of ***z*** that can be explained by ***W***.

We estimated the variance components of the above model using Restricted Maximum likelihood (REML [7]) which was implemented with a custom R-script that for optimization uses the bobyqa function of the minqa R-package [33]. The scripts used to fit variance components using REML are provided in the S1 File (see function fitREML).

### Partial least squares

We also estimated the proportion of variance of ***X*** explained by ***W*** (and the reciprocal when needed) by regressing ***X*** on ***W*** using the pls R-package [34].

X=1μ+Wβ+ε.

The ability of the pls regression to fit ***X*** depends on the number of components used. To determine the number of components, we first fitted the PLS regressions with *1*, *2*, *…*, *100* components in 10-fold cross-validation and evaluated the cross-validation prediction mean-square error of each of the resulting models. We then selected the number of components that led to the smallest mean-squared prediction error and fitted a PLS regression with that number of components to the entire data set. The R^2^ of the fitted model in the training data was used as an estimate of the proportion of variance of ***X*** that could be explained by ***W***. The fitPLS function provided in the script provides a wrapper to the plsr function which implements the procedure described above.

### Simulations

Both simulations were implemented using genotypes from the wheat data set.

#### Simulation 1

In the first simulation setting the input set was the wheat genotypes ***W***_599×1,279_ = {***w***_1_,…,***w***_1,279_}, and ***X*** = {***x***_1_,…,***x***_1,279_} was a noisy version of ***W*** obtained by adding Gaussian *iid* noise, $\delta_{i}∼N(0,I\sigma_{\delta }^{2})$, to the genotypes, ***x***_*i*_ = ***w***_*i*_+***δ***_*i*_ where *i = 1*,*…*, *1*,*279*. The columns of ***W*** were standardized to unit variance, and the noise variance ($\sigma_{\delta }^{2}$) was set such that the proportion of variance of ***x***_*i*_ explained by ***w***_*i*_ was equal to 0.1, 0.3, 0.5, 0.8, 0.9 and 1:

$$R_{X∼W}^{2}=\frac{Cov(x_{i},w_{i})}{Var(x_{i})}=\frac{Var(w_{i})}{Var(x_{i})}=\frac{1}{1+\sigma_{\delta }^{2}}.$$

We also consider a scenario where $R_{X∼W}^{2}=0$ (i.e., ***X*** was purely random noise).

We conducted 1,000 MC simulations (the input set (***W***) did not change across MC samples; however, the output set (***X***) varied across MC replicates due to the noise term), and, for each simulated data set, estimated the proportion of variance of ***X*** explained by regression on ***W*** using MC-ANOVA, Eigen-ANOVA, and the PLS method.

#### Simulation 2

We designed a second simulation to consider the case where one of the sets (***X***) was included in the other set (***W***). In this setting ***X***_599×*p*_ was generated by including *p (≤1,279)* of the 1,279 DNA-markers; we used values of *p* that led to the inclusion of 5%, 10%, 30%, 50%, 80%, 90%, and 95% of all the available DNA markers. Subsequently, ***W*** was formed by combining ***X*** with (*p-1*,*279*) columns filled with *iid* Gaussian random variables (***Z***): ***W***_599×1,279_ = [***X***_599×*p*_,***Z***_599×(*p*−1,279)_].

The columns of ***X*** and ***W*** were all centered and scaled to unit variance. Since ***Z*** is independent of ***X***, the proportion of variance of ***W*** explained by ***X*** equals *p*/1,279. On the other hand, the proportion of variance of ***X*** explained by **W** is one because ***X*** is included in ***W***. We conducted 1,000 MC simulations and, for each simulated data sets, we estimated $R_{X∼W}^{2}$ and $R_{W∼X}^{2}$ by regressing ***X*** on ***W*** and ***W*** on ***X***, respectively, using MC-ANOVA, Eigen-ANOVA, and the PLS method.

## Supporting information

S1 File Contains the scripts used to carry out the simulations and data analyses. (HTML)
