# Supplementary material for: ANOVA-HD: Analysis of variance when both input and output layers are high-dimensional
Source: PLoS One. 2020 Dec 14;15(12):e0243251. doi: 10.1371/journal.pone.0243251 (PMC7735570; doi:10.1371/journal.pone.0243251)
Supplement: S1 File — (HTML) [file pone.0243251.s001.html]

SCRIPTS


# SCRIPTS

#### G. de los Campos

# I. R-scripts

## I.1 Functions used for REML and PLS estimation

### REML

```
# Objective function
neg2_REML<-function(y,Xt,vars,d,n,nSuccess=1,nFailure=1){
    ###
    # y=U'eHat where is eHat=OLS residuals
    # Xt=X'U
    # vars=c(varE,varU)
    # d: eigenvalues
    # n: length(y)
    # objective function is -2*L2 (page 1 in http://www.aps.uoguelph.ca/~lrs/Animalz/lesson11/lesson11.pdf)
    # gustavoc@msu.edu
    ###     

    varE=(vars[1])
    varU=(vars[2])

        w<-d*varU+varE

        neg2_reml_1=sum(log(w))

        Xt<-scale(Xt,center=F,scale=1/sqrt(w))
        C=tcrossprod(Xt)
        neg2_reml_2<-log(det(C))

        neg2_reml_3<-sum((y^2)/w)

        h2=varU/(varU+varE)

        neg2LogPrior=-2* ( (nSuccess-1)*log(h2)+(nFailure-1)*log(1-h2))

        out<-neg2_reml_1+neg2_reml_2+neg2_reml_3#+neg2LogPrior
        return(out)
}

# Model fittting
fitREML<-function(y,EVD=NULL,K=NULL,n=length(y),X=matrix(nrow=n,ncol=1,1),
                  nSuccess=1,nFailure=1,computeHessian=F){
  ###
  # y: phenotype
  # EVD=eigen(G)
  # X: incidence matrix for fixed effects
  # gustavoc@msu.edu 11/30/2016
  ###
  if(is.null(EVD)){
      if(is.null(K)){
          stop('provide either K or its eigenvalue decomposition')
      }else{
          EVD=eigen(K)
      }
  }


  library(minqa)
  library(numDeriv)


  varY<-var(y)

  tmp<-rep(TRUE,length(EVD$values))#EVD$values>1e-5
  d=EVD$values[tmp]
  U=EVD$vectors[,tmp]

  eHat<-residuals(lsfit(y=y,x=X,intercept=F))
  y<-crossprod(U,eHat)
  Xt<-crossprod(X,U)

  fm<-bobyqa(par=varY*rep(.5,2),fn=neg2_REML,lower=rep(1e-5,2)*varY,upper=rep(1.5,1.5)*varY,y=y,Xt=Xt,d=d,n=n,
             nSuccess=nSuccess,nFailure=nFailure)

  estimates=c(fm$par,fm$par[2]/sum(fm$par))


  names(estimates)<-c('varE','varU','h2')
  out=list(estimates=estimates,logREML=-2*fm$fval)
 if(computeHessian){

   COV1=solve(hessian(fun=neg2_REML,x=fm$par,y=y,Xt=Xt,d=d,n=n,
             nSuccess=nSuccess,nFailure=nFailure))

  X=matrix(nrow=100000,ncol=2,rnorm(200000))%*%chol(COV1)
  X[,1]=X[,1]+estimates[1]
  X[,2]=X[,2]+estimates[2]

  COV=matrix(nrow=3,ncol=3,NA)
  COV[1:2,1:2]=COV1
  h2=X[,2]/rowSums(X)
  COV[3,3]=var(h2)
  COV[3,1]=COV[1,3]=cov(X[,1],h2)
  COV[3,2]=COV[2,3]=cov(X[,2],h2)

  out$SEs=sqrt(diag(COV))
  out$vcov=COV
 }
  return(out)
}
```

### PLS

```
fitPLS=function(Y,X,nComp){
  library(pls)
  timeIn=proc.time()[3]
   fm<-plsr(Y~X, ncomp=nComp,validation='CV')
   CV.R2=1-(fm$validation$PRESS)/(fm$validation$PRESS0)
   CV.R2=colMeans(CV.R2)

   nComp=which.max(CV.R2)
   EHat=fm$residuals[,,nComp]

   RSS0=sum(Y^2)
   RSS1=sum(EHat^2)
   R2=(RSS0-RSS1)/RSS0
  
  timeOut=proc.time()[3]

  out=c(nComp,timeOut-timeIn,R2)
  names(out)=c('nComp','seconds','R2')
  return(out)
}
```

**Example**

```
if(FALSE){
 library(BGLR)
 data(wheat)
 X=scale(wheat.X)
 G=tcrossprod(X)
 G=G/mean(diag(G))
 EVD=eigen(G)
 y=wheat.Y[,2]
 fitREML(y=y,EVD=EVD)
 
 fitPLS(Y=y,X=X,nComp=100)
 
}
```

**Note:** To run the scripts below you need to include the two functions above into a file `REML_PLS.R`

## I.2 Simulations

**Note**: The scripts presented below were run in a Linux cluster using a job array. The variable jobID was read into R from the command line. To test the scripts you can set `jobID=1`.

### Simulation 1

```
if(FALSE){ # change this to TRUE to read jobID from the command line
  args=(commandArgs(TRUE))
    for(i in 1:length(args)){
       eval(parse(text=args[[i]]))
    }
}else{
  jobID=4
}

 library(BGLR)
 
 data(wheat)
 X=scale(wheat.X)
 Gx=tcrossprod(X);Gx=Gx/mean(diag(Gx))
 
## Monte Carlo Study IID features ##############################################
 h2=c(0,.05,.1,.3,.5,.8,.9,.95,1)[jobID]
 nRep=3 # number of MC replicatesm set to 3 fpor code testing..
 nVectors=30 # number of random vectors used in the MC-method.

 R2_MC=rep(NA,nRep);
 names(R2_MC)=paste0('rep_',1:nRep)
 R2_EIGEN=R2_MC
 R2_PLS=R2_MC
 
 setwd('../output')

 for(j in 1:nRep){
    
        timeIn=proc.time()
        W=X;n=nrow(W);p=ncol(W)
        
        if(jobID==1){ # pure noise
                W=matrix(nrow=n,ncol=p,data=rnorm(n=n*p))
        }else{   # other scenarios
            for(k in 1:ncol(W)){
                W[,k]=X[,k]+rnorm(n=n,sd=sqrt(var(X[,k])*(1-h2)/h2))
            }
        }

        W=scale(W)
        Gw=tcrossprod(W);Gw=Gw/mean(diag(Gw))

        EVDw=eigen(Gw)
        EVDx=eigen(Gx)

        # MC-ANOVA
        SUM.ESTIMATES=0
        for(k in 1:nVectors){
            a=rnorm(ncol(W))
            y=as.vector(scale(W%*%a))
            Vy=var(y)
            h2Hat=fitREML(y=y,EVD=EVDx)$estimates['h2']
            SUM.ESTIMATES=SUM.ESTIMATES+h2Hat
        }
        
        R2_MC[j]=SUM.ESTIMATES/nVectors
   
        # EIGEN-ANOVA
        nV=length(EVDw$values)
        tmp=rep(NA,nV)
        for(k in 1:nV){
            y=as.vector(scale(EVDw$vectors[,k]))
            h2Hat=fitREML(y=y,EVD=EVDx)$estimates['h2']
            tmp[k]=h2Hat
        }
        R2_EIGEN[j]=sum(tmp*EVDw$values)/sum(EVDw$values)
        
        ## PLS
        W=scale(W)
        fm<-fitPLS(W,X,nComp=100)
      
        R2_PLS[j]=fm['R2']
          

        save(R2_MC,R2_EIGEN,R2_PLS,file=paste0('RESULTS_h2_',h2,'.RData'))

        timeOut=proc.time()
        message("==== h2=",h2," -> MC-rep=",j," ",round((proc.time()-timeIn)[3],2))
        message(round(mean(R2_MC[1:j]),3)," ",round(mean(R2_EIGEN[1:j]),3))
  }
```

### Simulation 2

```
if(FALSE){ # change this to TRUE to read jobID from the command line
 args=(commandArgs(TRUE))
 for(i in 1:length(args)){
    eval(parse(text=args[[i]]))
 }
}else{
  jobID=4
}
 library(BGLR)
 library(pls)

 data(wheat)
 X=scale(wheat.X)
 n=nrow(X)
 p=ncol(X)
 Gx=tcrossprod(X);Gx=Gx/mean(diag(Gx))

## Monte Carlo Study IID features ##############################################
 h2=c(.05,.1,.3,.5,.8,.9,.95,1)[jobID]
 nRep=3 # number of MC replicates
 nVectors=30 # number of random vectors used in the MC-method.

 R2_MC=matrix(NA,nrow=nRep,ncol=2);colnames(R2_MC)=c('W~X','X~W')
 names(R2_MC)=paste0('rep_',1:nRep)
 R2_EIGEN=R2_MC
 R2_PLS=R2_MC

 setwd('../output')
 dir.create(method)
 setwd(method)

 q=round(ncol(X)*h2)
 
 for(j in 1:nRep){
        timeIn=proc.time()
        
        W=scale(matrix(nrow=n,ncol=p,rnorm(n*p)))

          cols=sample(1:p,size=q,replace=F)
        X0=scale(X[,cols])
        W[,1:q]=X0
 
        Gw=tcrossprod(W);Gw=Gw/mean(diag(Gw))
          Gx=tcrossprod(X0);Gx=Gx/mean(diag(Gx))
        EVDw=eigen(Gw)
        EVDx=eigen(Gx)

        # MC-ANOVA
        
        # W~X
         SUM.ESTIMATES=0
         for(k in 1:nVectors){
            a=rnorm(ncol(W))
            y=as.vector(scale(W%*%a))
            Vy=var(y)
            h2Hat=fitREML(y=y,EVD=EVDx)$estimates['h2']
            SUM.ESTIMATES=SUM.ESTIMATES+h2Hat
         }
         R2_MC[j,'W~X']=SUM.ESTIMATES/nVectors

        # X~W
         SUM.ESTIMATES=0
         for(k in 1:nVectors){
            a=rnorm(ncol(X0))
            y=as.vector(scale(X0%*%a))
            Vy=var(y)
            h2Hat=fitREML(y=y,EVD=EVDw)$estimates['h2']
            SUM.ESTIMATES=SUM.ESTIMATES+h2Hat
         }
         R2_MC[j,'X~W']=SUM.ESTIMATES/nVectors

        # EIGEN-ANOVA
         # W~X        
         nV=length(EVDw$values)
         tmp=rep(NA,nV)
         for(k in 1:nV){
            y=as.vector(scale(EVDw$vectors[,k]))
            h2Hat=fitREML(y=y,EVD=EVDx)$estimates['h2']
            tmp[k]=h2Hat
         }
         R2_EIGEN[j,'W~X']=sum(tmp*EVDw$values)/sum(EVDw$values)

            # X~W
         nV=length(EVDx$values)
         tmp=rep(NA,nV)
         for(k in 1:nV){
            y=as.vector(scale(EVDx$vectors[,k]))
            h2Hat=fitREML(y=y,EVD=EVDw)$estimates['h2']
            tmp[k]=h2Hat
         }
         R2_EIGEN[j,'X~W']=sum(tmp*EVDx$values)/sum(EVDx$values)

         ## PLS
         R2_PLS[j,'X~W']=fitPLS(Y=X,X=W,nComp=150)
         R2_PLS[j,'W~X']=fitPLS(Y=W,X=X,nComp=150)
         
         save(R2_MC,R2_EIGEN,R2_PLS,file=paste0('RESULTS_h2_',h2,'.RData'))

        timeOut=proc.time()
        message("==== h2=",h2," -> MC-rep=",j," ",round((proc.time()-timeIn)[3],2))

  }
```

### Chicken genomes I (results for traits with normal weights on all the UHD genotypes)

```
library(BGData)
source('REML_PLS.R')

load('../../data/X.RData')
setwd('../output')

p=ncol(X)
Gx=getG(X)
EVDx=eigen(Gx)
nVectors=sum(EVDx$values>1e-5)


panels=list()
arraySize=c(.5,1,2,5,10,50)*1000

i=length(arraySize)
 chunkSize=ceiling(ncol(X)/arraySize[i])
 INI=floor(chunkSize/2)
 END=ncol(X)-floor(chunkSize/2)
 panels[[i]]=floor(seq(from=INI,to=END,length=arraySize[i]))

 panels[[i-1]]=panels[[i]][rep(c(F,F,T,F,F),times=10000)]
 panels[[i-2]]=panels[[i-1]][rep(c(T,F),times=5000)]
 panels[[i-3]]=panels[[i-2]][rep(c(F,T,F,T,F),TIMES=1000)]
 panels[[i-4]]=panels[[i-3]][rep(c(F,T),times=1000)]
 panels[[i-5]]=panels[[i-4]][rep(c(T,F),times=500)]

### EIGEN
EVD=list()
Z=list()

for(i in 1:length(arraySize)){
    W=X[,panels[[i]]]
    G=tcrossprod(scale(W,scale=F,center=T))
    G=G/mean(diag(G))
    EVD[[i]]=eigen(G)
    print(i)
}

R2vectors=matrix(nrow=nVectors,ncol=length(panels))
colnames(R2vectors)=paste0('N-SNPs=',arraySize)
rownames(R2vectors)=paste0('vec_',1:nrow(R2vectors))

for(i in 1:ncol(R2vectors)){
    for(j in 1:nrow(R2vectors)){
        y=EVDx$vectors[,j]
        fm=fitREML(y=y,EVD=EVD[[i]])
        R2vectors[j,i]=fm$estimates['h2']
        message(i,"  ",j)
    }
}

EIGEN_ANOVA=colSums(R2vectors*EVDx$values[1:nVectors])/sum(EVDx$values)


## MC-METHOD
nRep=50
MC_ANOVA=matrix(nrow=nRep,ncol=length(panels))
colnames(MC_ANOVA)=paste0('N-SNPs=',arraySize)
rownames(MC_ANOVA)=paste0('rep_',1:nRep)

for(i in 1:ncol(MC_ANOVA)){
    for(j in 1:nrow(MC_ANOVA)){
        
        y=X%*%rnorm(ncol(X))
        fm=fitREML(y=y,EVD=EVD[[i]])
        MC_ANOVA[j,i]=fm$estimates['h2']
        message(i,"  ",j)
    }
}

save(EIGEN_ANOVA,MC_ANOVA,file='GLOBAL.RData')
```

### Chicken genomes II (results for traits with 5,10,50, and 500 causal variants)

```
library(BGData)
source('REML_PLS.R')
load('../../data/X.RData')
p=ncol(X)


panels=list()
arraySize=c(.5,1,2,5,10,50)*1000

i=length(arraySize)
 chunkSize=ceiling(ncol(X)/arraySize[i])
 INI=floor(chunkSize/2)
 END=ncol(X)-floor(chunkSize/2)
 panels[[i]]=floor(seq(from=INI,to=END,length=arraySize[i]))

 panels[[i-1]]=panels[[i]][rep(c(F,F,T,F,F),times=10000)]
 panels[[i-2]]=panels[[i-1]][rep(c(T,F),times=5000)]
 panels[[i-3]]=panels[[i-2]][rep(c(F,T,F,T,F),TIMES=1000)]
 panels[[i-4]]=panels[[i-3]][rep(c(F,T),times=1000)]
 panels[[i-5]]=panels[[i-4]][rep(c(T,F),times=500)]

print(arraySize)
print(str(panels))

nReps=500

### Monte Carlo Method
EVD=list()
Z=list()

for(i in 1:length(arraySize)){
        W=X[,panels[[i]]]
        G=tcrossprod(scale(W,scale=F,center=T))
        G=G/mean(diag(G))
        EVD[[i]]=eigen(G)
        print(i)
}


tmp=matrix(nrow=nReps,ncol=length(panels))
colnames(tmp)=paste0('N-SNPs=',arraySize)
rownames(tmp)=paste0('rep_',1:nrow(tmp))

### MC-Method
library(BGData)

setwd('../output')

nQTN=c(5,10,50,500)
R2.MC=list()

for(h in 1:length(nQTN)){
        R2.MC[[h]]=matrix(nrow=nReps,ncol=length(arraySize))
        colnames(R2.MC[[h]])=arraySize

        for(i in 1:nrow(R2.MC[[h]])){
                for(j in 1:ncol(R2.MC[[h]])){
                        
                        colsX=sample(1:p,size=nQTN[h])
                        W=X[,colsX]
                        
                        ## Monte-Carlo method (30 random vectors)
                        tmp=0
                        for(k in 1:30){                               
                                a=rnorm(nQTN[h])
                                y=W%*%a
                                fm=fitREML(y=y,EVD=EVD[[j]])
                                tmp=tmp+fm$estimates['h2']/30
                        }
                        R2.MC[[h]][i,j]=tmp
                }
                if(i%%10==0){ message(h," ",i) }
        }
        save(R2.MC,file='R2_MC.RData')
}

quit(save='no')
```

### Multi-omic, breast cancer data (TCGA)

```
source('REML.R')
load("/mnt/research/quantgen/datasets/TCGA/edData/omic_blocks.rda",verbose=T)
load("/mnt/research/quantgen/datasets/TCGA/edData/inputData.RData",verbose=T)

#Getting primary tumors from breast.
tmp <- which(sample.type=="Primary" & cancer.type =="BRCA")
XGE<-  scale(GE[tmp,]); 
XME <- scale(DM[tmp,]); 
XCNV<- scale(CNV[tmp,]);


library(BGData)
G_GE=getG(XGE)
G_ME=getG(XME)
G_CNV=getG(XCNV)

EVD_GE=eigen(G_GE)
EVD_ME=eigen(G_ME)
EVD_CNV=eigen(G_CNV)

nRep=3000
MC=matrix(nrow=nRep,ncol=6)
colnames(MC)=c('GE~M','GE~CNV','M~GE','M~CNV','CNV~GE','CNV~M')

EIGEN=matrix(ncol=ncol(MC),nrow=nrow(EVD_GE$vectors),0)
colnames(EIGEN)=colnames(MC)


#1&2# ge~met
for(i in 1:nRep){
 x=XGE%*%rnorm(ncol(XGE))
 fm=fitREML(y=x,EVD=EVD_ME)
 MC[i,'GE~M']<-fm$estimates['h2']

 fm=fitREML(y=x,EVD=EVD_CNV)
 MC[i,'GE~CNV']=fm$estimates['h2']

 x=XME%*%rnorm(ncol(XME))
 fm=fitREML(y=x,EVD=EVD_GE)
 MC[i,'M~GE']<-fm$estimates['h2']

 fm=fitREML(y=x,EVD=EVD_CNV)
 MC[i,'M~CNV']=fm$estimates['h2']

 x=XCNV%*%rnorm(ncol(XCNV))
 fm=fitREML(y=x,EVD=EVD_GE)
 MC[i,'CNV~GE']<-fm$estimates['h2']

 fm=fitREML(y=x,EVD=EVD_ME)
 MC[i,'CNV~M']=fm$estimates['h2']

 message(i)

}

for(i in 1:nrow(EIGEN)){

  if(EVD_GE$values[i]>1e-8){
   #GE~M
      fm=fitREML(y=EVD_GE$vectors[,i],EVD=EVD_ME)
      EIGEN[i,'GE~M']=fm$estimates['h2']    

   #GE~CNV
      fm=fitREML(y=EVD_GE$vectors[,i],EVD=EVD_CNV)
    EIGEN[i,'GE~CNV']=fm$estimates['h2'] 
  }


  if(EVD_ME$values[i]>1e-8){
   #M~GE
    fm=fitREML(y=EVD_ME$vectors[,i],EVD=EVD_GE)
    EIGEN[i,'M~GE']=fm$estimates['h2']

   #M~CNV
    fm=fitREML(y=EVD_ME$vectors[,i],EVD=EVD_CNV)
    EIGEN[i,'M~CNV']=fm$estimates['h2']
  }

  if(EVD_CNV$values[i]>1e-8){
   #CNV~GE
    fm=fitREML(y=EVD_CNV$vectors[,i],EVD=EVD_GE)
    EIGEN[i,'CNV~GE']=fm$estimates['h2']
   #CNV~ME
    fm=fitREML(y=EVD_CNV$vectors[,i],EVD=EVD_ME)
    EIGEN[i,'CNV~M']=fm$estimates['h2']
  }
}

EValues=cbind(EVD_GE$values,EVD_ME$values,EVD_CNV$values)
colnames(EValues)=c('values_ge','values_me','values_cnv')

save(EValues,MC,EIGEN,file='../output/results.RData')
```
